# Artificial womb: opportunities and challenges for public health

**DOI:** 10.1097/JS9.0000000000000208

**Published:** 2023-03-24

**Authors:** Yashita Khulbe, Shiva Gupta, Binish Javed, Ahmad Neyazi, Bijaya K. Padhi

**Affiliations:** a King George’s Medical University, Lucknow, Uttar Pradesh; b Atal Bihari Vajpayee Institute of Medical Sciences and Dr. Ram Manohar Lohia Hospital, New Delhi India; c Afghanistan Center for Epidemiological Studies, Herat, Afghanistan; d Department of Community Medicine and School of Public Health, Postgraduate Institute of Medical Education and Research, Chandigarh, India

According to the WHO, 15 million babies are born prematurely each year, of which 1 million die due to complications. The need to reduce neonatal mortality has culminated in the development of artificial amnion and placenta technology, commonly known as the ‘Artificial Womb’, which provides an environment for the ectogestation of the foetus. Ever since 1958, when Westin *et al*. developed the first artificial womb by cannulation of umbilical vessels, this technology has shown remarkable potential for improvement of clinical outcomes in critically preterm children. Currently, working models of the technology include EXTra-uterine Environment for Neonatal Development (EXTEND) by Children’s Hospital of Philadelphia, Ex-Vivo uterine Environment (EVE) by Tohoku University, and University of Western Australia^1^. The world’s first artificial womb facility – EctoLife – was launched on 9 December 2022 by a filmmaker and science communicator based in Berlin, Germany^2^.

The ‘Womb’ has been designed to simulate all the necessary physiological mechanisms during gestation. Gaseous exchange is performed via extracorporeal membrane oxygenation. A pumpless arteriovenous circuit is deployed that drives blood exclusively from the foetal heart and is combined with a low-resistance oxygenator. Waste disposal is carried out through dialysis. A polyethylene film-based biobag is used, which is responsible for sterility, size adjustment, and fluid and space volume efficiency. It can be modified to mimic the dimensions and shape of the uterus with more accuracy, thus providing an analogous alternative to the real womb^3^.

Considering the incidence and mortality rates of premature deliveries throughout the world, artificial womb technology (AWT) is no less than a boon. AWT provides the innate environment of a human womb, thereby reducing respiratory struggle incompatible with the premature lung of the foetus. The impervious, sealed design mimicking the amniotic cavity also reduces the risk of infection. It can be a suitable alternative in cases of placental insufficiency, which can lead to preterm labour and intrauterine growth retardation. The health benefits also extend to pregnant women, as this technology provides a safe alternative to high-risk pregnancies and will alleviate the risks of concluding a full-term pregnancy. Women frequently experience pregnancy-related concerns including anaemia, hypertension, mental health issues, and viral infections. Some of these may be fatal to both the mother and the child, but could be prevented with AWT.

The benefits on the social front are also very uplifting. AWT enables infertile couples to conceive a child and become the real biological parents of their children. Moreover, it raises a good proportion of the responsibility of carrying a baby, which is otherwise entirely instilled upon the female. Allows both parents to share equal responsibilities even before the child is fully born, raising equity amongst both genders^4^. This will help women both physically and will mentally and be more independent. AWT is a helpful alternative to surrogacy for same-sex couples, females who have undergone hysterectomy or infertile women.

However, there are still issues that need to be resolved. Technology currently aids in the development of the foetus only between 13 weeks and before complete gestation, that is, 38 weeks. As it cannot facilitate embryo to foetal development, it is bound to be preceded by a surgical extraction of the foetus, which may be associated with complications^4^. The models do not possess the natural ability of the placenta to reduce pulsatile flow from the umbilical artery to laminar flow in the umbilical vein^5^. Furthermore, there is no collective understanding of the long-term psychological and psychological effects of the womb on parents and future offspring, considering the change it will bring to the concept of ‘giving birth’.

Given the multitude of possible applications, the AWT is not without ethical concerns. The main considerations for the foetus include the consequentialism of medical interventions and their legal status, while those for parents include the extent of their autonomy in decision-making on behalf of the infant, impact on maternal bonding, cost-effectiveness, and accessibility. Even societal arguments about the acceptability of ectogenesis and regulation of abortion pose a question that should be answered before the release of this technology.

A potential, though remotely possible, application of the technology is ‘complete ectogenesis’ – complete gestation outside the human body. This will greatly affect the substantiated human involvement during gestation, making it an extracorporeal event and thus completely transforming the conventional notion of pregnancy.

## Ethical approval

Not applicable.

## Sources of funding

None.

## Author contribution

The authors declare that they have no financial conflict of interest with regard to the content of this report.

## Conflicts of interest disclosure

There are no conflicts of interest.

## Guarantor

A. Neyazi.

## Data statement

We have not collected any primary data for this research. The authors confirm that the data supporting the findings of this study are available within the article and/or its supplementary materials.
